# Suture-tape augmentation of anterior cruciate ligament reconstruction: a prospective, randomised controlled trial (STACLR)

**DOI:** 10.1186/s13063-023-07127-0

**Published:** 2023-03-24

**Authors:** Lachlan Huntington, Andrew Griffith, Libby Spiers, Rebecca Pile, Lachlan Batty, Scott Tulloch, Phong Tran

**Affiliations:** Department of Orthopaedic Surgery, Western Health, Footscray Hospital, Level 1 South, Gordon St, Footscray, Melbourne, VIC 3011 Australia

**Keywords:** Orthopaedics, ACL, Anterior cruciate ligament, Reconstruction, Randomised controlled trial, RCT, Suture tape, Augmentation, Arthrometer, PROMs, GNRB, Tape

## Abstract

**Background:**

Anterior cruciate ligament (ACL) reconstruction aims to restore anteroposterior and rotatory stability to the knee following ACL injury. This requires the graft to withstand the forces applied during the process of ligamentisation and the rehabilitative period. We hypothesise that the use of suture tape augmentation of single bundle ACL reconstruction (ACLR) will reduce residual knee laxity and improve patient-reported outcomes at 2-year follow-up. We will conduct a 1:1 parallel arm single-centre randomised controlled trial comparing suture tape augmented ACLR to standard ACLR technique.

**Methods:**

The study design will be a parallel arm 1:1 allocation ratio randomised controlled trial. Sixty-six patients aged 18 and over waitlisted for primary ACLR will be randomised. Patients requiring osteotomy and extra-articular tenodesis and who have had previous contralateral ACL rupture or repair of meniscal or cartilage pathology that modifies the post-operative rehabilitation will be excluded. The primary outcome measure will be the side-to-side difference in anterior tibial translation (measured on the GNRB arthrometer) at 24 months post-surgery. GNRB arthrometer measures will also be taken preoperatively, at 3 months and 12 months post-surgery. Secondary outcomes will include patient-reported outcome measures (PROMs) collected online, including quality of life, activity and readiness to return to sport, complication rates (return to theatre, graft failure and rates of sterile effusion), examination findings and return to sport outcomes. Participants will be seen preoperatively, at 6 weeks, 3 months, 12 months and 24 months post-surgery. Participants and those taking arthrometer measures will be blinded to allocation.

**Discussion:**

This will be the first randomised trial to investigate the effect of suture-tape augmentation of ACLR on either objective or subjective outcome measures. The use of suture-tape augmentation in ACLR has been associated with promising biomechanical and animal-level studies, exhibiting equivalent complication profiles to the standard technique, with initial non-comparative clinical studies establishing possible areas of advantage for the technique. The successful completion of this trial will allow for an improved understanding of the in situ validity of tape augmentation whilst potentially providing a further platform for surgical stabilisation of the ACL graft.

**Trial registration:**

Australia New Zealand Clinical Trial Registry ACTRN12621001162808. Universal Trial Number (UTN): U1111-1268-1487. Registered prospectively on 27 August 2021

**Supplementary Information:**

The online version contains supplementary material available at 10.1186/s13063-023-07127-0.

## Administrative information

Note: the numbers in curly brackets in this protocol refer to SPIRIT checklist item numbers. The order of the items has been modified to group similar items (see http://www.equator-network.org/reporting-guidelines/spirit-2013-statement-defining-standard-protocol-items-for-clinical-trials/).

**Table:** 

Title {1}	Suture-tape augmentation of anterior cruciate ligament reconstruction: a prospective, randomised controlled trial (STACLR).
Trial registration {2a and 2b}.	This study was registered prospectively in the Australia New Zealand Clinical Trial registry on 27/8/21 under Trial Number ACTRN12621001162808. Universal Trial Number (UTN): U1111-1268-1487.
Protocol version (1)	Protocol version 1.3, Date: 30.08.2021
Funding {4}	This study is both self-funded and supplemented by post ethics approval for funding by Arthrex Global Investigator-Initiated Research Grant, as well as the Victorian Orthopaedic Foundation Grant.
Author details {5a}	^1^Dr Lachlan Huntington, ^1^Dr Andrew Griffith, ^1^Ms Libby Spiers, ^1^Ms Rebecca Pile, ^1^Mr Lachlan Batty, ^1^Mr Scott Tulloch, ^1^Prof Phong Tran. ^1^Department of Orthopaedic Surgery, Western Health, Footscray Hospital, Level 1 South, Gordon St, Footscray, Melbourne, VIC 3011, Australia
Name and contact information for the trial sponsor {5b}	Sponsor: Western Health Sponsor Contact Details: Mr Bill Karanatsisios, Research Program Director, Western Health Office for Research Level 3 Office for Research, Western Centre for Health & Research Education (WCHRE), Sunshine Hospital, 176 Furlong Road, St Albans, Vic 3021
Role of sponsor {5c}	The sponsor and any funders will have no role of the study design, collection of data, analysis or interpretation of study data, decision to submit for publication. The study sponsor will ensure compliance with ethical delivery of the trial in accordance with Good Clinical Practice standards.

## Introduction

### Background and rationale {6a}

The anterior cruciate ligament (ACL) acts as the primary static constraint to anterior translation and internal rotation of the tibia [1]. ACL rupture is a common knee injury, affecting 0.03–3.67% of athletes annually [2], and most frequently the result of non-contact pivoting sporting injury [2]. ACL rupture in a young person has been established to result in significant loss of participation in sport, the need for reconstructive surgery and prolonged rehabilitation [3], whilst long-term osteoarthritis risk can be increased [3, 4]. Historically, reconstruction with autograft has been the most common pathway to return to sport [5]. Recent reports suggest, however, that return to sport rates are as low as 55% [6], whilst only 40% make a complete functional recovery [7]. Secondary to this, graft failure remains a significant problem, particularly in at-risk populations such as younger and female athletes [8, 9] and those who yield smaller autograft widths [9] at surgical reconstruction. Given these current limitations of ACL reconstruction, Nagelli and Hewett have suggested that return to sport should be delayed to up to 2 years postoperatively [10]. Successful restoration of anterior tibial translatory stability with ACL reconstruction is dependent on the capacity of the ACL graft to withstand appropriate loads during rehabilitation, and the return to the sporting period. As such, synthetic augmentation of native autograft or indeed wholesale replacement of autograft has been of interest for many years. Primarily, these augments were the Ligament Augmentation and Reconstruction System (LARS) prosthesis, the Kennedy Ligament Augmentation Device (LAD), the Leeds-Keio device, the Dacron device and GORE-TEX devices and have been primarily utilised as complete substitutes for autograft [11], with their use as graft augments also reported [12, 13]. However, failure rates are up to 33%, particularly in earlier devices, whilst more recent devices such as the LARS ligament have been reported to be associated with higher rates of persistent effusion and foreign body synovitis [14].

Recently, the use of suture tape (a modified suture composed of non-absorbable, braided polyethylene/polyester suture) acting as a ‘seatbelt’ for in situ autograft has been proposed [15–17]. It differs from historical graft augments in its ‘addition’ to autograft rather than replacement of it, and its successful use in ligamentous reconstruction and repair in other joints is encouraging [18–20]. Notably, the use of suture tape may improve initial graft stability and protect the graft prior to ligamentisation. Significantly, it has been studied to exhibit improved biomechanical stability relative to non-augmented grafts [21–23]. Additionally, animal studies have reported equivalent functional, arthroscopic and histological findings between augmented and unaugmented knees [24–27]. Importantly, rates of persistent synovitis, rates of graft incorporation and incidence of adverse histological findings are not reported to differ between suture-tape augmented grafts and control grafts among animal models [24, 27]. A recent human model report of 36 ACL reconstructions with suture tape augmentation recorded no persistent joint effusion or evidence of synovitis [28]. However, prospective clinical data is lacking. A 2019 retrospective comparative study of 60 knees exhibited reduced postoperative pain, as well as improved change from baseline PROMs at 1 year after all-inside ACLR with suture-tape augmentation [29]. However, this study is limited both in its retrospective design and its lack of objective assessment of knee stability. In contrast to this study, Parkes et al. have reported on 36 suture tape-augmented reconstructions, finding no significant differences in rates of return to sport, mean IKDC scores or post-operative examination findings compared with a 2:1 matched cohort [28]. Whilst these studies offer promise regarding possible benefits of suture tape augmentation, they lack a control group and complete clinical assessment to the standard 2-year mark post-operatively. Significantly, the influence of graft augmentation upon residual anterior knee laxity after reconstruction of the ACL has not been reported across the literature. This study aims to address these deficits.

The primary research objective is to determine whether the use of suture-tape augmentation of a primary hamstring autograft ACLR improves post-operative residual knee laxity. At present, the literature surrounding suture tape augmentation of ACLR is primarily biomechanical and animal model-based, with two retrospective cohort, non-randomised studies reporting subjective outcomes published. This study aims to collect a higher level of evidence, as well as objective measures of post-operative knee laxity among suture tape ACLR patients.

The null hypothesis of this study is that there will be no significant difference in side-to-side residual anterior knee laxity, as measured with the GNRB arthrometer, as compared with the contralateral knee at 2 years post-operatively. Secondary outcomes will be graft failure, complication rates, clinical examination findings and patient-reported outcome measures (PROMs) including Knee Osteoarthritis and Outcomes Score Quality of Life subscale (KOOS-QOL) [30], International Knee Documentation Committee scale (IKDC) [31], Marx activity scale [32], EQ-5D-5L scale [33] and ACL-Return to Sport Index (ACL-RSI) [34] scores measured at 2 years post-operatively.

The study aims to investigate whether suture-tape augmented anterior cruciate ligament reconstruction has superior results to non-augmented reconstructions.

## Objectives {7}

### Primary objective

The objective of this study is to compare residual anterior knee laxity after primary hamstring autograft ACLR with or without suture tape (ST) augmentation, as measured by the GNRB ligament arthrometer at 2 years post-operatively. The primary outcome measure will be the difference between operative and non-operative limbs (side-to-side), between groups, at 2 years.

### Secondary objectives

The secondary objectives are to compare PROMs, complication rates, return to sport rates and examination findings between SA and ST ACLR. The PROMs assessed will include the Marx activity scale, ACL RSI, IKDC, the KOOS QOL and the EQ5D-5L scale. Complications assessed will include post-operative pain levels, graft failure rates, contralateral knee ACL rupture, return to theatre and findings including rates of particulate-related synovitis, sterile effusion, persistent effusion and symptomatic arthrofibrosis of the knee. Return to sport data will include rates of return to sport, timing of return to sport and rates of return to the previous level of the sport. Examination findings assessed will include the presence of effusion, knee range of motion and the presence and grade of the Lachman test, anterior drawer and pivot shift tests.

## Trial design {8}

This study is a randomised controlled single-blind interventional 2-arm parallel-group superiority trial utilising 1:1 allocation ratio comparing ACLR with suture tape augmentation (ST-ACLR) to standard hamstring ACLR technique (ACLR) with femoral adjustable cortical fixation and tibial interference screw. This study is a superiority study with the hypothesis that ACL reconstruction with suture tape augmentation is superior to standard ACL reconstruction technique. All patients meeting inclusion criteria will be randomised to a treatment arm in the operating room following confirmation of exclusion criteria at arthroscopy. All components of the study will be undertaken at a single institution. All patients will be followed up to 2 years post-operatively.

## Methods: participants, interventions and outcomes

### Study setting {9}

This study will be conducted across three subsidiary hospitals, within a single major metropolitan Australian academic hospital (site details available on ANZ Trial Registry). All patients will be recruited from the adult orthopaedic outpatient clinic. A total of 66 participants will be recruited and randomised to a treatment arm. Owing to the nature of the randomisation model, greater than 66 patients may be recruited to reach the eventual target randomisation number. All data will be collected within Australia.

### Eligibility criteria {10}

All inclusion criteria will be assessed by the treating surgeon and associate investigators.

Participants can be included if they are:

- Waitlisted for ACLR with either of the associated investigators (S.T or L.B). Waitlisting is based on evidence of complete ACL rupture, based on clinical assessment and MRI imaging. Patients waitlisted will also have the appropriate lifestyle indications to warrant surgical reconstruction of the ligament.
- Able to give informed consent and to participate fully in the interventions and follow-up procedures.
- Aged 18 and over.
- Concomitant meniscal and/or osteochondral pathology can be included.
- Planned for surgery using an ipsilateral hamstring tendon autograft

Patients will be excluded if they:

- Have had a previous ACL reconstruction on the ipsilateral knee.
- Have had a previous ACL injury to the non-operative knee.
- Are of a developmental age where the presence of open physes would otherwise alter the surgical technique utilised.
- Have grade 2 or 3 medial collateral ligament (MCL)/lateral collateral ligament (LCL) injury, associated posterior cruciate ligament (PCL)/ posterolateral corner (PLC) injury that requires surgical intervention.
- Have inflammatory arthritis.
- Are pregnant.
- Have an articular cartilage defect requiring treatment that would alter the post-operative rehabilitation protocol and timelines.
- Have a meniscal injury requiring treatment that would alter the post-operative rehabilitation protocol and timelines (i.e. meniscal root or bucket handle tear repair).
- Have an ACL re-rupture risk significant enough to warrant the addition of an osteotomy or deformity corrective procedure or lateral extraarticular tenodesis.

### Who will take informed consent? {26a}

Participants will undergo assessment and provide written informed consent for ACLR surgery with their treating surgeon or one of the orthopaedic department’s trainee surgeons in consultation with the treating surgeon. Patients waitlisted for primary ACLR with either of the Associate Investigators (ST or LB) will be approached and offered participation in the study. Patients will either be approached in person or via phone after their appointment. All consent for trial involvement will be performed by an informed member of the research team. The patient will be advised that they have the right to privacy and any information obtained in connection with this project and that could identify them will remain strictly confidential. Information will only be disclosed with the patient’s permission, except as required by law. They will be informed that at the time that in the unlikely event that a patient is deceased, any original paper records kept will be treated according to standardised hospital policies. Given the intention-to-treat basis of this trial, data already collected will be included within the trial analysis. Information may be used in a deidentified manner within presentations and publications in peer-reviewed medical journals. In accordance with relevant Australian and/or Victorian privacy and other relevant laws, the patient will have the right to access the information collected and stored by the researchers. Patients will be informed that they are free to refuse participation, and if they decide or withdraw at any time, they will not compromise their future medical care. In either case, they will be given a physical or digital copy of the participant information and consent form (PICF) and offered the opportunity to ask questions of any member of the research team. They will be then sent (or given) a digital consent form via REDCAP to sign electronically, which will be then stored in our institution’s secure REDCap platform. A copy of the signed consent form will also be sent to the participant.

### Additional consent provisions for collection and use of participant data and biological specimens {26b}

Participants will undergo extended consent for use of study information in ancillary studies emanating from this trial. Participants will be advised that the results of this study may be utilised in a de-identified manner within publications or scientific research presentations.

### Interventions

#### Explanation for the choice of comparators {6b}

This study will compare ACL reconstruction with hamstrings autograft with (ST-ACLR) and without (ACLR) suture tape augmentation. The suture tape will be looped through the proximal femoral button. Both procedures are standard care at our institution. The surgical technique has been decided at the discretion of the senior surgeons.

#### Intervention description {11a}

##### Graft fixation

Fixation will be performed with an adjustable suspensory ACL TightRope® 2 RT device (Arthrex, Naples, FL, USA) on the femoral side and a PEEK interference screw (Arthrex, Naples, FL, USA) on the tibial side with a diameter the same size as the tibial tunnel.

##### Graft harvest and preparation

The semitendinosus and gracilis tendons will be harvested from the ipsilateral knee with a tendon harvester. Each tendon will be doubled over the ACL TightRope® 2 RT device (Arthrex, Naples, FL, USA) and the free ends sutured together with 2-Fibreloop suture (Arthrex, Naples, FL, USA) to create a 4-strand hamstring graft. If the graft diameter is less than 7mm in females or 7.5 mm in males, both tendons will be tripled to create a 6-strand construct. The length of the graft may be whipstitched with a 1 Vicryl suture to tubularise and compress the graft.

##### Femoral tunnel preparation

An anatomic single-bundle reconstruction will be performed. The ACL femoral footprint will be identified and a point between the AM and PL bundles will be selected, erring towards the AM bundle. The position will be confirmed from the medial portal. A Spade Tip guide wire (Arthrex, Naples, FL, USA) will be passed via the anteromedial portal with the knee in maximum flexion and the tunnel length measured off the Spade Tip drill guide. A femoral reamer corresponding to the graft femoral diameter will be drilled via the anteromedial portal over the SpadeTip wire (Arthrex, Naples, FL, USA) to a depth of 25 to 30mm. A shuttling suture will be placed.

##### Tibia tunnel preparation

The tibial footprint will be identified and any residual tibial stump will be preserved where possible. An Arthrex ACL aimer set to 55° will be passed via the medial portal to facilitate the passage of a guide wire through the mid-point of the tibial ACL attachment. The full tibial tunnel will be created with a cylindrical reamer corresponding with the graft tibial diameter [26].

##### Graft passage, tensioning and fixation technique

The femoral button sutures will be passed through the femoral tunnel using the shuttling suture. The cortical suspensory button will be passed and confirmed to have flipped with a toggle test and reference to a marking on the tightrope corresponding to the femoral tunnel length and a second mark 7 mm closer to the graft. The graft will then be docked into the femoral tunnel, 5 mm short of the tunnel depth by shortening the adjustable loop. Two markings on the graft corresponding to the tunnel depth and 5 mm short of the tunnel depth as measured from the femoral end of the graft will be used to assess the amount docked into the femoral tunnel. The tibial side of the graft is then cycled 15 times with maximal manual tension applied. Following this, a proprietary “fish scale” tensioning device (Arthrex Inc, Naples, FL) is used to apply tension to the graft at 80N and an interference screw (PEEK; Arthrex Inc.) is placed over a nitinol wire with the knee at 0° of flexion with a posterior force on the tibia. The knee is then cycled through range of motion to ensure full range. The graft re-tensioned from the femoral side at 30° of flexion.

##### Suture tape augmentation technique

Patients randomised to the SA arm will use the Tightrope 2 femoral cortical button, which has a single strand of Fibertape looped through the proximal Tightrope to be run alongside the graft to serve as an augment. The femoral button and graft will then be docked into the femoral tunnel, 5 mm short of the tunnel depth by shortening the adjustable loop in a similar fashion to the standard ACL reconstruction group. The tibial side of the graft is then cycled 15 times with maximal manual tension applied. Secondary tibial fixation of the suture tape will be performed before the tibial graft, with a 4.75-mm SwiveLock anchor in full extension. A guide pin was drilled 1.5 cm distal to the tibial tunnel to a depth of 20mm and overrreamed with a 3.4-mm diameter reamer and 4.75-mm tap. Ensuring the free ends of the Fibertape are separate from the tibial-sided graft sutures, a haemostat is then placed underneath the FiberTape limbs to ensure it is not tighter than the graft. The suture tape is then fixed with a 4.75-mm SwiveLock anchor with the knee in maximal knee extension. The graft will be tensioned at 0° of knee flexion with a proprietary “fish scale” tensioning device used to apply tension to the graft at 80N, and a posterior force on the tibia. The tibial side of the graft will then be secured with an interference screw (PEEK; Arthrex Inc.), with the suture tape running alongside the graft through the tibial tunnel, with tension on the graft only. The knee is then cycled through a range of motion to ensure the full range is present. In order to ensure greater tension on the graft than the tape, the femoral side of the graft is then re-tensioned to ensure fully docked within the femoral tunnel at 30° of knee flexion.

##### Closure

Closure is performed after irrigation and haemostasis in a layered fashion.

#### Criteria for discontinuing or modifying allocated interventions {11b}

Owing to the nature of the intervention, being a surgical technique and use of specific implant, and the timing following randomisation, it is not anticipated that any participants will be discontinued from allocated intervention following randomisation. If a participant suffers graft failure or contralateral ACL knee rupture, then analysis via GNRB assessment may be changed. Participants may withdraw from the study without citing a reason, but reasons may include the participant has chosen to withdraw, postoperative graft rupture or failure or the participant has experienced an adverse event. If a patient indicates that they wish to withdraw, this will be recorded in the REDCap project, and they will not be asked to complete any further research-only assessments. They will be reassured that they will continue to receive care and any subsequent management at Western Health. If a patient withdraws from the study, data collected to this point will remain within the study for analysis unless the participant specifically requests otherwise.

#### Strategies to improve adherence to interventions {11c}

All follow-up, short of one interaction, will be performed in conjunction with standard surgical care. All PROMs will be collected by easily accessible medium, including text message or email contact. This study will be co-ordinated by a dedicated research assistant in order to give participants the time to ask and have answered questions with regard to trial or concomitant care, as well as to ensure the workload of trial delivery is adequately met. The research team will remain in contact with all participants and encourage their attendance at standard care appointments where data collection will occur via SMS reminders and phone calls as required.

#### Relevant concomitant care permitted or prohibited during the trial {11d}

Post-operatively, all patients will undergo standardised medical and rehabilitative protocols according to the Fowler Kennedy Physiotherapy following ACL Reconstruction Protocol. All patients will receive day 1 physiotherapy and standard post-discharge management including wound review at 2 weeks, with clinical reviews at 6 weeks, 3 months and 12 months. Participants will be offered institutional physiotherapy or be free to pursue physiotherapy through an independent provider. Participants will not be asked to modify any of their medication or treatment relating to other medical conditions.

Return to sport will not be permitted until criteria stipulated by the Fowler Kennedy protocol are met, as agreed by the senior investigators. As stipulated by the protocol, return to sport is initiated in a graduated format from the 6–9-month mark, provided the operative knee is without pain or effusion during, or after functional sports-related training drills. Lower extremity function scores (LEFS) should be 76 points or greater at this point in rehabilitation. The LEFS is a self-reported questionnaire used to evaluate the functional status of an individual with a lower extremity musculoskeletal dysfunction. The individual must also be able to demonstrate the appropriate strength and endurance needed for their specific sport.

### Provisions for post-trial care {30}

All participants will be offered routine medical and surgical post-trial care commensurate with their condition at the discretion of the treating surgeon. There will be no compensation offered to participants either for their involvement in the study. This is outlined in the participant information and consent form.

### Outcomes {12}

#### Primary outcome

The primary outcome will be the side-to-side difference in anterior tibial translation as measured on the GNRB arthrometer at 2 years post-operatively, between groups. Maximum anteroposterior tibial translation at 134 N will be recorded on both the operative and non-operative knees pre-operatively and post-operatively at 3, 12 and 24 months. The goals of ACL reconstruction are to restore sagittal and rotatory stability to the knee and to prevent secondary injuries such as meniscal tears, and progression to osteoarthritis [35]. Common means of assessing this is by comparison of pre- and post-operative subjective Lachman manoeuvre and anterior drawer; however, these are often imprecise and subjective [36]. Consequently, devices to accurately measure anterior tibial translation have been developed, most popularly the KT-1000 ™ manual arthrometer (MEDmetric®, San Diego, USA) [37]. However, this device has been criticised for poor inter- and intra-observer reliability, and as such, newer computerised models such as the GNRB (Genourob, Laval, France) have been developed, with improved reliability [38]. Studies have utilised these arthrometers to compare between anterior cruciate ligament surgical techniques, such as graft types [39, 40], assessing post-operative outcomes [41], as well as analysing residual joint laxity after ligament reconstruction [42]. This outcome has been selected due to its ability to assess whether the addition of suture tape in ACLR results in knee stability that is more similar to the contralateral uninjured knee than standard ACLR technique. Anterior tibial translation and knee laxity may vary from individual to individual, and for this reason, side-to-side difference has been chosen as the primary outcome, allowing the contralateral knee to be a within-participant baseline measure, reducing the variability in outcome owing to between participant differences.

#### Secondary outcomes

Secondary outcomes are fourfold and will include (i) PROMs, (ii) return to sport rates, (iii) complications and (iv) examination findings.

i) PROMs will be recorded preoperatively and post-operatively at 6 weeks, 3 months, 12 months and 24 months. PROMs collected will include the following:
  1. EQ5D-5L: The EuroQol EQ-5D-5L is a validated, generic, self-reported outcome measure covering five health domains that are used to facilitate the calculation of quality-adjusted life years (QALYs) in health economic evaluations. The original EQ-5D questionnaire contained three response options within each of five health domains (mobility, self-care, usual activities, pain/discomfort and anxiety/depression) [33]. More recently, the EQ-5D-5 L has been developed to overcome problems with ceiling effects and to improve sensitivity [43]. The 5 L version consists of the same five domains as the original but with five response options.
  2. The International Knee Documentation Committee (IKDC) score: The IKDC is a knee-specific patient outcome questionnaire often used after ACL reconstruction [31]. It is a subjective tool that provides an overall patient score (0–100), often interpreted as a measure of function, where higher scores represent a greater function. It aims to address three categories: symptoms, activity and overall function, and has shown adequate test-retest reliability and good construct validity in patients with issues at the knee [44].
  3. Knee injury and Osteoarthritis Outcome Score Quality of Life sub-scale (KOOS-QOL): This subscale is a 4-item questionnaire designed to evaluate the knee-specific quality of life, has been utilised among ACL surgical trials [30] and has been established to have validity, responsiveness and reliability in patients undergoing ACLR [45].
  4. ACL return to sport index (ACL-RSI): Psychological readiness to return to sport will be evaluated with the ACL-RSI. It is a 12-point questionnaire validated to evaluate a patient’s confidence and readiness to return to sport [34] and exhibits high internal validity and structural consistency [46]. Given the theoretical increased stability offered by suture tape augmentation, particularly in the early rehabilitative period, it follows that patients treated with this may experience improved perception of knee function and subsequent confidence in rehabilitation and sporting activity across the early postoperative period.
  5. Self-reported pain: We will record postoperative pain levels and long-term knee pain scores utilising 10-point visual analogue scale pain scores (VAS) where 0 is no pain and 10 is the worst pain imaginable.
ii) Complications:
  We will record complications and adverse events including wound complications, infection rates, return to surgery, e.g. meniscal tears, graft failure, contralateral knee ACL rupture, residual effusion, rates of symptomatic arthrofibrosis of the knee and second look arthroscopy findings and the rates of sterile effusion. These will be recorded upon review of each patient’s medical record at 2 years.
iii) Return to sport and activity:
  Return to sport and activity will be assessed via a self-reported questionnaire delivered concomitantly with PROMs preoperatively and post-operatively at 6 weeks, 3 months, 12 months and 24 months. Return to activity will be evaluated through the Marx activity scale, a four-item activity rating scale which has been shown to have excellent retest reliability in patients with knee injuries [32]. Participants will be asked to record on a scale of 0–4 how often they are able to perform each of running, cutting, deceleration and pivoting in their most active state across the preceding 12 months. A maximum score is 16 points. Participants will also be asked about their dichotomous return to sport status, their expectations regarding timing of return to sport, return to preinjury level of sport and the time postoperatively to return to sporting activity. These questions, along with the full set of pre-operative and post-operative PROMS, are included in Additional file 1.
iv) Examination findings
  Examination findings will be taken preoperatively at examination under anaesthesia and post-operatively at 6 weeks, 3 months and 12 months. Examination findings including the range of motion (ROM), the presence of knee effusion, Lachmans, pivot shift and anterior drawer testing will be recorded. All assessments will be undertaken by lead surgeons S.T, L.B.

### Participant timeline {13}

**Fig. 1:**
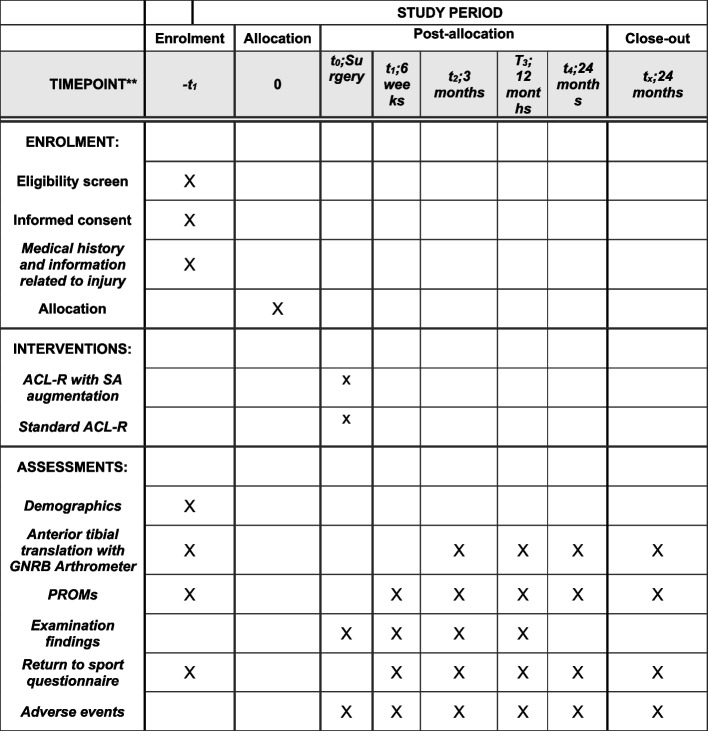
SPIRIT figure summarising outcomes and assessment schedule

**Fig. 2:**
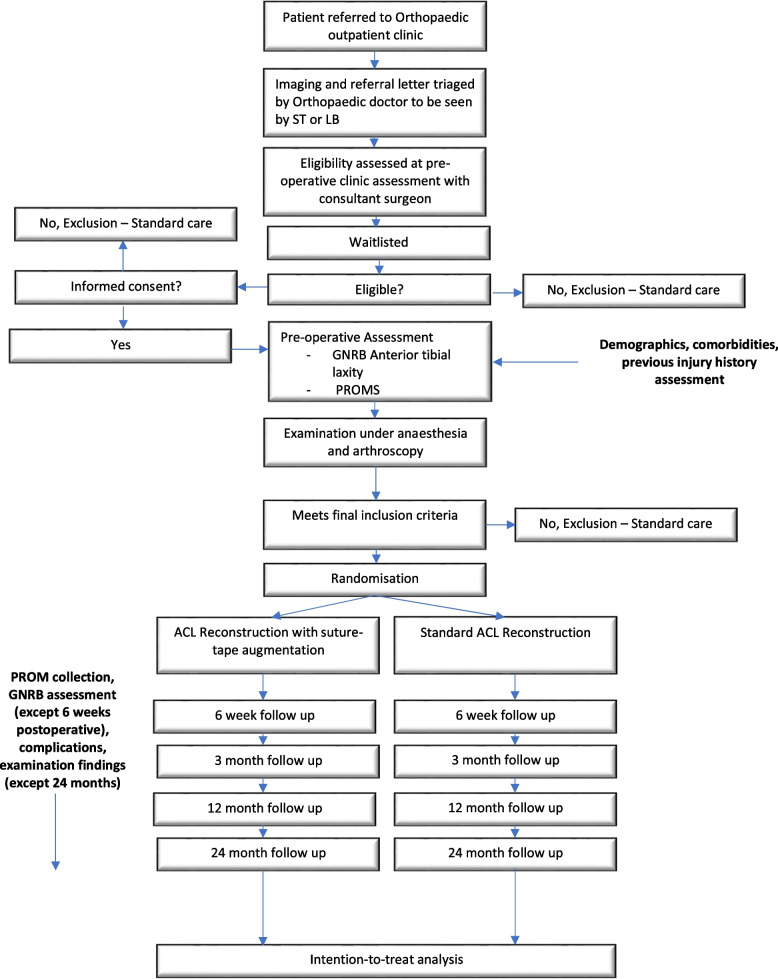
Trial flow pathway

Participants will be recruited from the orthopaedic outpatient clinic at our institution. Participants will be eligible for assessment if they are referred to either of the associated investigators’ clinic (L.B or S.T) and are consented for ACLR. Eligibility for trial participation based on inclusion and exclusion criteria will then be assessed. Patients who are eligible will be offered a discussion about the study and the opportunity to ask questions in line with best-practice informed consent. Data will be collected pre-operatively and post-operatively at four time points (6 weeks, 3 months, 1 year and 2 years) and will include arthrometric measure of anterior tibial translation on the GNRB arthrometer and PROM-based data, as well as return to sport data, complication rates and examination findings (Fig. 1). Participants will then be asked to complete an online questionnaire about their injury, preoperative return to sport expectations and PROMs. Following this, they will be referred for routine preoperative rehabilitation and undergo preoperative GNRB assessment with a member of the research team. Demographic data will be extracted from their medical record with the participant’s consent at this time. Initial examination findings will be recorded at examination under anaesthesia preceding arthroscopy. Final evaluation of eligibility criteria will occur during arthroscopy. Those patients who have consented for involvement in the study and are deemed eligible during surgery will be randomised using the REDCap platform and allocated to one of the trial arms (ACLR or ST-ACLR) and blinded to the allocation (Fig. 2).

Postoperatively, all GNRB measurements will be taken at the same standard time points to routine surgical follow-up by a member of the research team, or a member of the physiotherapy unit familiar with GNRB protocols. All PROMs and return to sport data will be recorded via online surveys directly into the REDCAP database. Follow-up surveys will be conducted via automated email service at the corresponding time point. All subsequent examinations will be conducted by lead surgeons S.T and L.B with data entered directly into a study data collection report form (paper version), and then manually entered into the REDCAP database. There will be only one time point (2 years) at which patients will be required to attend follow-up (for GNRB testing) without the need for a standard clinical follow-up as per standard practice; otherwise, patients will be within the hospital for their standard clinical follow-up at the time of testing.

### Sample size {14}

All statistical methods and power analysis were developed through consultation with an independent statistician. Power analysis was performed with G*power 3.1 Software. To achieve a minimum 80% statistical power with an alpha value of 0.05, power analysis based on a hypothesised mean residual side-to-side difference of 2 mm of anteroposterior tibial translation yielded a sample size of *n*=48 (24 per group) utilising an expected independent *t*-test [47–50]. Although previously published KT-1000 data was utilised for power analysis, it has been shown that the GNRB exhibits similar absolute measures in side-to-side anterior tibial translation to KT-1000 utilised by experienced observers, whilst having reduced scatter around the mean [38]. This would mean that utilising KT-1000 published data to establish a power analysis would be a conservative method, and likely over-estimate the required sample size to detect significant differences.

Secondary power analysis based on 2-year IKDC total scores yielded a total sample size of 48 (*n*=24 in each group) to detect significant differences with 80% power and an alpha value of 0.05 with an anticipated independent sample two-tailed *t*-test. This is based on a previous retrospective study of suture-tape ACL reconstruction which reported a mean change from baseline IKDC of 56 ± 17 in SA-augmented ACLR, compared with 38 ± 25 in conventional ACLR [29, 51]. Secondly, previous studies evaluating the IKDC [51] which reported that a change score of 12.8 points would be sufficient to detect differences between those who have and have not had improved knee outcomes. Power analysis yielded a sample size of 48. Dropout rates are expected to be 30% based on institutional experience, owing to loss to follow-up and contralateral ACL rupture and graft failure, and as such 66 patients will be recruited. This is expected to take approximately 1–2 years of recruitment based on historical institutional caseloads.

### Recruitment {15}

Recruitment will be conducted continuously across patients eligible until the target randomised sample size is achieved. Based on institutional caseloads, it is anticipated this will take 1–2 years. A dedicated member of the research team will be tasked with recruitment on clinic days, such that all prospective participants are given appropriate opportunity to understand the study before and during involvement.

## Assignment of interventions: allocation

### Sequence generation {16a}

The randomisation design is a computer-generated permuted single-blind block randomisation. The randomisation sequence will be developed by an independent statistician, with all other research team members blinded to the randomisation sequence. Participants will be randomised using permuted block randomisation which allows for a better guarantee of equal-sized treatment groups [52] whilst protecting against prediction of allocation towards the end of the recruitment period. Randomisation will be allocated in a 1:1 fashion, whereby patients will undergo either standard ACLR or suture tape-augmented ACLR. Randomisation will be stratified according to age greater than 25 years.

### Concealment mechanism {16b}

The centrally managed, blinded randomisation model will ensure allocation concealment and prevent selection bias. The allocation sequence will be stored within the REDCap database and inaccessible to all but the statistician, ensuring concealment. Following randomisation, the allocation details will be displayed on the web-based system for each participant, and an automated email will be sent to members of the research team to ensure appropriate documentation in the medical record.

### Implementation {16c}

The allocation sequence will be developed by an independent statistician. It will be integrated into the REDCap automated randomisation platform and will be blinded to all other investigators. Enrolment of participants will be overseen by the lead surgeons, and associate investigators ST and LB, and will be carried out by associate investigators LH and EN. Randomisation and assignment to the intervention arm will be performed intraoperatively, following assessment and management of concomitant cartilage and meniscal injuries by the associate investigators LB and ST.

## Assignment of interventions: blinding

### Who will be blinded {17a}

Trial participants will be blinded to the allocation arm for the duration of the study. GNRB arthrometric measurements are inherently low in bias, secondary to the computer-generated application of force to the posterior tibia; however, technicians taking the measures will be blinded to allocation arm. Owing to the nature of examination assessments, the best technician to carry these out is the same as the treating surgeon and thus blinding is not appropriate in this setting. The questionnaire-based outcomes are patient-reported and therefore also assessor-blinded.

### Procedure for unblinding if needed {17b}

As the surgeon and only outcome assessors are not blinded, unblinding will not be required.

## Data collection and management

### Plans for assessment and collection of outcomes {18a}

All data will be entered into a custom web-based REDCap database accessible by study research staff only. All GNRB data will be transposed from automated Genuroub software outputs to custom-designed data entry forms in the preoperative and postoperative setting by a member of the research team specifically trained in both software. PROMs and return to sport data will be collected by a single questionnaire and entered directly via online surveys by the participant. All validated PROM scores are cited above and can be found in the original citation; all non-validated questionnaires are included in the Additional file 1 of this document. Examination findings will be entered specifically into the medical record of each participant, and a study-specific data collection form and transposed into the web-based database.

### Plans to promote participant retention and complete follow-up {18b}

PROMs and return to sport data will be collected by online survey, deliverable by email or text message with the aims to increase follow-up rates among a technologically inclined patient cohort. All follow-up examinations and GNRB assessments will be performed concurrently with follow-up surgical appointments until the 12-month mark in order to reduce the study burden on the individual. If a participant withdraws from the study, or suffers graft failure, no further data will be collected; however, data collected up until this point will be included in the study and absent data will be analyzed according to intention to treat principles.

### Data management {19}

All data will be stored on a secure institutional REDCap server in a database custom-built for this study. Validation of data fields will be built into this platform where relevant, as well as the use of “required” fields to minimise missing data. Double data entry will not be possible.

### Confidentiality {27}

The research information will be re-identifiable. All participants will be assigned a study ID. A data re-identification key file will be stored as an encrypted file separate to the file containing the data. This will be a password-protected file stored on the hospital server. Only the research team can match the participant’s name to their code number, if it is necessary to do so. The Principal Investigator will be responsible for the secure storage of the data collected in this project. Any hard copy data will be identified by a study ID number only and kept secure in a locked filing cabinet within a locked office. Only named researchers will have access to all data collected in this project. Electronic datasets will be stored securely within the institutional server as a password-protected excel file/on the institutional REDCap platform.

### Plans for collection, laboratory evaluation and storage of biological specimens for genetic or molecular analysis in this trial/future use {33}

No biological specimens will be collected as part of this study.

## Statistical methods

### Statistical methods for primary and secondary outcomes {20a}

Side-to-side difference in anterior tibial translation (the primary outcome), overall ACL-RSI, EQ5D-5L, Marx activity scales, IKDC and KOOS-QOL will be calculated as means, with measures of dispersion reported as standard deviation. Differences between groups will be reported as mean differences, with dispersion reported with confidence intervals. Data normality will be assessed with the Shapiro-Wilk test for continuous parametric assessment. Between-group comparisons, such as the primary outcome of mean difference in GNRB anteroposterior laxity at 2 years, will be assessed with independent samples *t*-tests. If data is found to be non-parametric in distribution, the Wilcoxon rank sum test will be utilised. Comparison of two dichotomous variables, such as return to sport rates and complication rates, will be performed with Fischer’s exact test. Repeated measures such as GNRB measures and mean PROMs will be assessed using mixed effects linear analysis. All tests are two-sided. A *p* value of <0.05 will be considered the cut-off for statistical significance.

### Interim analyses {21b}

Interim analysis will be performed 1 year after the surgery of the final randomised participant. There will be no formal criteria for trial termination; however, if it is observed that the intervention is associated with significant harm to subsequently enrolled participants, then consideration of trial termination will be conducted.

### Methods for additional analyses (e.g. subgroup analyses) {20b}

Subgroup analysis will be explored to identify possible treatment effect modifying baseline factors such as age, sex, return to sport expectations and pre-operative ipsilateral knee laxity as measured on the GNRB arthrometer.

### Methods in analysis to handle protocol non-adherence and any statistical methods to handle missing data {20c}

All principal analyses will be based on the intention-to-treat principle, analysing participants in the groups to which they are randomised. Missing data will be quantified and if possible multiple imputation will be used; otherwise, simple imputation will be used. Owing to the nature of enrolment and randomisation in this study, it is unlikely that a participant will be randomised and subsequently not receive the intervention, and thus adherence does not apply directly to the intervention arm.

### Plans to give access to the full protocol, participant-level data and statistical code {31c}

The protocol will be registered at the Australia New Zealand Clinical Trials Registry (ANZCTR), where specifics not present in the current publication may be reviewed.

## Oversight and monitoring

### Composition of the coordinating centre and trial steering committee {5d}

The Principal Investigator, the research assistant/co-ordinator and at least one other Investigator (Internal Trial Monitoring Committee) will meet at least monthly to monitor the progress of the trial to discuss study progress and procedures, adverse events and any other issues,

### Composition of the data monitoring committee, its role and reporting structure {21a}

The Principal Investigator will act as the data manager for the trial. The research team will meet as above in lieu of an official Data Monitoring Committee. Direct access to the data will be granted to authorised representatives from the sponsor, host institution or ethics board for monitoring and/or audit to ensure compliance with regulations.

### Adverse event reporting and harms {22}

Participant safety will be ensured via standard institutional protocols. The use of the GNRB is established to be safe in pre-operative [53] and early post-operative patients [42], with no adverse events reported, and this will be expressed verbally to patients prior to participation. Adverse events are defined as medical occurrences that may or may not have a causal relationship with the surgical treatment administered. Any adverse events identified will be recorded and reviewed regularly by the Internal Trial Monitoring Committee. The Principal Investigator will determine causality. The number and type of adverse events (AEs) will be recorded up to 24 months. Any AEs will be noted on the REDCap database. These events will also be reviewed by the participants’ medical and surgical teams (who are not a part of the investigative team) as part of standard care.

Serious adverse events (SAE) are defined as any untoward and unexpected medical occurrence that results in death, is life-threatening, requires hospitalisation or prolongation of existing inpatients’ hospitalisation, results in persistent or significant disability or incapacity, is a congenital anomaly or birth defect, or is any other important medical condition which, although not included in the above, may require medical or surgical intervention to prevent one of the outcomes listed. All SAEs will be reported to the approving ethics board within 72 h of the investigators becoming aware of them. SAEs that may be expected as part of the surgical interventions and that do not need to be reported to the HREC are complications of anaesthesia or surgery (wound infection, bleeding or damage to adjacent structures such as nerves, tendons and blood vessels, delayed wound healing, and thromboembolic events, femoral or tibial fracture).

### Frequency and plans for auditing trial conduct {23}

Auditing of trial conduct will be performed at a frequency and depth as determined by the local ethics board, independent of investigators and research team.

### Plans for communicating important protocol amendments to relevant parties (e.g. trial participants, ethical committees) {25}

All modifications to study protocols, following approval with the local ethics board, will be documented as amendments in the ANZ clinical trial registry. All participants having previously signed consent forms prior to change in protocol will be notified by email including the detail of the change and its impact on them and offered an opportunity to review a new written consent. If the change will not impact the participants’ involvement in the study, a change in the statistical methods, or a reduction in the frequency of recording of SAEs, or a change in study personnel, as an example, would not impact the participants’ involvement in the trial and thus they will not be notified of these changes.

### Dissemination plans {31a}

By signing the consent form, the participants give their permission to allow the de-identified data generated by this research to be shared/discussed with the local institutional orthopaedic unit and those working within it. Information may be used in publication in peer-reviewed medical journals. The results may also be presented at relevant national or international meetings and conferences. The information from this study will be disseminated within presentations or publications related to the present study, or within research related to this study.

## Discussion

This manuscript reports on the methodological design of the STACLR trial (Suture Tape Augmentation of Anterior Cruciate Ligament Reconstruction), the first randomised design prospective trial comparing suture tape-augmented ACL reconstruction to standard ACL reconstruction in adult patients. In this study, 2-year objective knee laxity, subjective patient-reported outcomes, complications and return to sport rates will be compared in patient treated with tape-augmented ACL reconstruction to those without. The hypothesis of the present study is that suture tape augmentation of ACL reconstruction will result in reduced side-to-side anterior tibial laxity at 2 years post-operative. Secondary hypotheses include an improvement in PROMs and equivalent complication profiles in tape-augmented grafts.

There are several biomechanical studies comparing tape-augmented reconstruction to standard technique, whilst several animal studies and a scattering of retrospective nonrandomised clinical studies exist, all of which provide an established concept and lay the platform for future research. The most devastating complication of ACL reconstruction may be graft failure; however, owing to the sample size demanded, the present study will not feasibly investigate this, and it is important to note this when interpreting results. In a retrospective cohort study of tape augmentation in ACL reconstruction, Parkes et al. [28] in a post hoc analysis reported a sample size of 1290 to adequately power for graft failure. Nevertheless, on principle, the action of tape augmentation will be to increase the biomechanical strength of the graft, a property that has been established by author groups including Noonan et al. [23] and Lai et al. [54], where tape-augmented grafts have higher load to failure and reduced elongation under cyclic loading. It follows that this may result in reduced laxity in post-operative knees compared with the standard ACL technique. Therefore, the role of tape augmentation in increasing biomechanical strength is to protect the graft during maturation and protect those grafts at higher risk of failure. Observed reduced knee laxity post-operatively is a well-utilised measure in evaluating the return of anteroposterior stability after ACL reconstruction [19–22]. Notably, early laxity has been associated with poor outcomes including increased risk of graft failure, reduced length of athletic career, permanently increased knee laxity and lower return to function scores [55]. Therefore, the use of an arthrometric measure, namely the GNRB anteroposterior laxity (selected on the basis of its purported superiority over the KT-1000), is an efficient method of assessing the efficacy of tape-augmentation in achieving its biomechanical goal in an achievable sample size.

Given the previous pitfalls of synthetic graft augmentation [11], skeptics of the technique may cite stress shielding or synovial complications such as sterile effusion as drawbacks to the approach. Animal studies, however, have evidence that despite the biomechanical protection of cyclic elongation, histological evidence of graft remodeling occurs [24, 26], signifying that with the appropriate tensioning of the tape in full extension, stress shielding may be avoided. Similarly, Smith et al. 29 have reported equivalent rates of synovitis and ongoing effusion in tape-augmented and non-augmented canine knees at 6 months. These early findings purporting reduced complication profiles observed in other synthetic graft models establish the need for further, prospective clinical research. This study aims to address some of the above outstanding questions remaining regarding suture tape augmentation of ACL reconstruction.

## Trial status

The current protocol is Version 1.3, dated 30 August 2021. Recruitment commenced on 03 March 2022. Recruitment is anticipated to finish on 30 June 2024.

## Supplementary Information

**Additional file 1.**

## Data Availability

All study personnel will have access to the final trial dataset.

## Abbreviations

ACL: Anterior cruciate ligament
ACLR: Anterior cruciate ligament reconstruction
SA: Suture tape-augmented anterior cruciate ligament reconstruction
ST: Standard anterior cruciate ligament reconstruction (non-augmented)
GNRB: Genurob anterior tibial translation ACL arthrometer
EQ5D-5L: EuroQuol 5 Dimension-5L
KOOS-QOL: Knee injury and Osteoarthritis Outcome Score Quality of Life subscale
IKDC: The International Knee Documentation Committee scale
ACL RSI: The Anterior Cruciate Ligament Return to Sport Index scale
VAS: Visual Analogue Scale
STACLR: Suture Tape Augmentation of Anterior Cruciate Ligament Reconstruction
SAE: Serious adverse event
ROM: Range of movement
PROM: Patient-related outcome measures
ANZCTR: Australia New Zealand Clinical Trials Registry
RCT: Randomised controlled trial
