# Supplementary material for: Suture-tape augmentation of anterior cruciate ligament reconstruction: a prospective, randomised controlled trial (STACLR)
Source: Trials. 2023 Mar 24;24:224. doi: 10.1186/s13063-023-07127-0 (PMC10037835; doi:10.1186/s13063-023-07127-0)
Supplement: Supplementary file 1 — Additional file 1. [file 13063_2023_7127_MOESM1_ESM.zip › 2021.093 RMH 73668 PostoperativePROMS_ACLRCT 08022021_ESM.pdf]

Postoperative PROMS

Study ID ________________

Hi [registration_arm_1][first_name],

Please answer these questions relating to your [registration_arm_1][side] knee operation on the [registration_arm_1][opdate].

If you are having difficulty viewing the questions on your mobile phone, please use a computer to complete them.

What is todays date? ________________

Please confirm if your previously listed email address ([registration_arm_1][email]) is still the best contact email?
- ◯ Yes
- ◯ No

What is the best email address to contact you on? ________________

Please confirm if your previously listed number [registration_arm_1][mobilenumber] remains your best contact number?
- ◯ Yes
- ◯ No

(Include Area Code)

Please list your new best contact number? ________________

What is your current height (cm) ________________

What is your current weight (kgs) ________________

Have you suffered a re- injury (complete rupture diagnosed by MRI or orthopaedic surgeon since the time of your operation) of your ACL in your [registration_arm_1][side] reconstructed knee?
- ◯ Yes
- ◯ No

How did you re-rupture your ACL? ________________

What date did you suffer your re-rupture? ________________

Since your operation on your [registration_arm_1][side] knee on [registration_arm_1][opdate], have you suffered an ACL rupture on your other knee?
- ◯ Yes
- ◯ No

Have you returned to full participation in sport?	◯ Yes<br>◯ No
What date did your return to full sporting participation?	__________________________________
Have you returned to your pre-injury level of sport?	◯ Yes<br>◯ No
Do you expect to be able to return to the same level of activity you were participating in prior to your [registration_arm_1][side] knee injury after recovering from your knee injury?	◯ Yes<br>◯ No - to a lower level<br>◯ No - I do not be expect to be able to return to activity at all
How long do you expect before you are able to return to such level of activity? (Months, from the day of your surgey)	__________________________________

EQ5D

Please indicate which statement best describes your own health state today.

Mobility	◯ I have no problems in walking about<br>◯ I have slight problems walking about<br>◯ I have moderate porblems in walking about<br>◯ I have severe problems in walking about<br>◯ I am unable to walk about
Self-care	◯ I have no problems washing or dressing myself<br>◯ I have slight problems washing or dressing myself<br>◯ I have moderate porblems washing or dressing myself<br>◯ I have severe problems washing or dressing myself<br>◯ I am unable to washing or dressing myself
Usual activities (eg work, study, housework, family or leisure activities)	◯ I have no problems doing my usual activities<br>◯ I have slight problems doing my usual activities<br>◯ I have moderate porblems doing my usual activities<br>◯ I have severe problems doing my usual activities<br>◯ I am unable to doing my usual activities
Pain/Discomfort	◯ I have no pain or discomfort<br>◯ I have slight pain or discomfort<br>◯ I have moderate pain or discomfort<br>◯ I have severe pain or discomfort<br>◯ I have extreme pain or discomfort
Anxiety/Depression	◯ I am not anxious or depressed<br>◯ I am slightly anxious or depressed<br>◯ I am moderately anxious or depressed<br>◯ I am severely anxious or depressed<br>◯ I am extremely anxious or depressed

We would like to know how good or bad your health is TODAY. The scale is numbered from 0 to 100.

100 means the best health you can imagine.
0 means the worst health you can imagine.

Please indicate on the scale how your health is TODAY.

0 50 100

(Place a mark on the scale above)

How much pain do you have in your [registration_arm_1][side] knee today?

0 50 100

(Place a mark on the scale above)

Marx activity scale

Please indicate how often you performed each activity in your healthiest and most active state, in the past year.

Running: running while playing a sport or jogging

- ◯ Less than one time in a month
- ◯ One time in a month
- ◯ One time in a week
- ◯ 2 or 3 times in a week
- ◯ 4 or more times in a week

Cutting: changing directions while running

- ◯ Less than one time in a month
- ◯ One time in a month
- ◯ One time in a week
- ◯ 2 or 3 times in a week
- ◯ 4 or more times in a week

Decelerating: coming to a quick stop while running

- ◯ Less than one time in a month
- ◯ One time in a month
- ◯ One time in a week
- ◯ 2 or 3 times in a week
- ◯ 4 or more times in a week

Pivoting: turning your body with your foot planted while playing a sport; For example: skiing, skating, kicking, throwing, hitting a ball (golf, tennis, squash), etc.

- ◯ Less than one time in a month
- ◯ One time in a month
- ◯ One time in a week
- ◯ 2 or 3 times in a week
- ◯ 4 or more times in a week

Marx Total

____

ACL RSI

Please mark which number best describes you in relation to the descriptors.

Are you confident that you can perform at your previous level of sport participation?
(0 = not at all confident, 10 = fully confident)

◯ 0 ◯ 1 ◯ 2 ◯ 3 ◯ 4 ◯ 5 ◯ 6 ◯ 7 ◯ 8 ◯ 9 ◯ 10

Do you think you are likely to re-injure your knee by participating in your sport?
(0 = extremely likely, 10 = not likely at all)

◯ 0 ◯ 1 ◯ 2 ◯ 3 ◯ 4 ◯ 5 ◯ 6 ◯ 7 ◯ 8 ◯ 9 ◯ 10

Are you nervous about playing your sport?
(0 =extremely nervous, 10 = not at all nervous)

◯ 0 ◯ 1 ◯ 2 ◯ 3 ◯ 4 ◯ 5 ◯ 6 ◯ 7 ◯ 8 ◯ 9 ◯ 10

Are you confident that your knee will not give way by playing your sport?
(0 = not at all confident, 10 = fully confident)

◯ 0 ◯ 1 ◯ 2 ◯ 3 ◯ 4 ◯ 5 ◯ 6 ◯ 7 ◯ 8 ◯ 9 ◯ 10

Are you confident that you could play your sport without concern for your knee?
(0 = not at all confident, 10 = fully confident)

◯ 0 ◯ 1 ◯ 2 ◯ 3 ◯ 4 ◯ 5 ◯ 6 ◯ 7 ◯ 8 ◯ 9 ◯ 10

Do you find it frustrating to have to consider your knee with respect to your sport?
(0 = extremely frustrating, 10 = not frustrating at all)

◯ 0 ◯ 1 ◯ 2 ◯ 3 ◯ 4 ◯ 5 ◯ 6 ◯ 7 ◯ 8 ◯ 9 ◯ 10

Are you fearful of re-injuring your knee by playing your sport?
(0 = extremely fearful, 10 = no fear at all)

◯ 0 ◯ 1 ◯ 2 ◯ 3 ◯ 4 ◯ 5 ◯ 6 ◯ 7 ◯ 8 ◯ 9 ◯ 10

Are you confident about your knee holding up under pressure?
(0 = not at all confident, 10 = fully confident)

◯ 0 ◯ 1 ◯ 2 ◯ 3 ◯ 4 ◯ 5 ◯ 6 ◯ 7 ◯ 8 ◯ 9 ◯ 10

Are you afraid of accidentally injuring your knee by playing sport?
(0 = extremely afraid, 10 = not at all afraid)

◯ 0 ◯ 1 ◯ 2 ◯ 3 ◯ 4 ◯ 5 ◯ 6 ◯ 7 ◯ 8 ◯ 9 ◯ 10

Do thoughts of having to go through surgery and rehabilitation prevent you from playing your sport?
(0 = all of the time, 10 = none of the time)

○ 0 ○ 1 ○ 2 ○ 3 ○ 4 ○ 5 ○ 6 ○ 7 ○ 8 ○ 9 ○ 10

Are you confident about your ability to perform well at your sport?
(0 = not at all confident, 10 = fully confident)

○ 0 ○ 1 ○ 2 ○ 3 ○ 4 ○ 5 ○ 6 ○ 7 ○ 8 ○ 9 ○ 10

Do you feel relaxed about playing your sport?
(0 = not at all relaxed, 10 = fully relaxed)

○ 0 ○ 1 ○ 2 ○ 3 ○ 4 ○ 5 ○ 6 ○ 7 ○ 8 ○ 9 ○ 10

ACL RSI Total

____

IKDC SUBJECTIVE KNEE EVALUATION FORM

What is the highest level of activity that you can perform without significant knee pain?

○ Very strenuous activities like jumping or pivoting as in basketball or soccer
○ Strenuous activities like heavy physical work, skiing or tennis
○ Moderate activities like moderate physical work, running or jogging
○ Light activities like walking, housework or yard work
○ Unable to perform any of the above activities due to knee pain

During the past 4 weeks, or since your injury, how often have you had pain?
(0 = Never and 10 = Constant)

○ 0 ○ 1 ○ 2 ○ 3 ○ 4 ○ 5 ○ 6 ○ 7 ○ 8 ○ 9 ○ 10

If you have pain, how severe is it?
(0 = No pain and 10 = worst pain imaginable)

○ 0 ○ 1 ○ 2 ○ 3 ○ 4 ○ 5 ○ 6 ○ 7 ○ 8 ○ 9 ○ 10

During the past 4 weeks, or since your injury, how stiff or swollen was your knee?

○ Not at all
○ Mildly
○ Moderately
○ Very
○ Extremely

What is the highest level of activity you can perform without significant swelling in your knee?

○ Very strenuous activities like jumping or pivoting as in basketball or soccer
○ Strenuous activities like heavy physical work, skiing or tennis
○ Moderate activities like moderate physical work, running or jogging
○ Light activities like walking, housework or yard work
○ Unable to perform any of the above activities due to knee swelling

During the past 4 weeks, or since your injury, did your knee lock or catch?

○ Yes ○ No

What is the highest level of activity you can perform without significant giving way in your knee?

◯ Very strenuous activities like jumping or pivoting as in basketball or soccer
◯ Strenuous activities like heavy physical work, skiing or tennis
◯ Moderate activities like moderate physical work, running or jogging
◯ Light activities like walking, housework or yard work
◯ Unable to perform any of the above activities due to giving way of the knee

SPORTS ACTIVITES:

What is the highest level of activity you can participate in on a regular basis?

◯ Very strenuous activities like jumping or pivoting as in basketball or soccer
◯ Strenuous activities like heavy physical work, skiing or tennis
◯ Moderate activities like moderate physical work, running or jogging
◯ Light activities like walking, housework or yard work
◯ Unable to perform any of the above activities due to giving way of the knee

How does your knee affect your ability to:

	Not difficult at all	Minimally difficult	Moderately difficult	Extremely difficult	Unable to do
a. Go up stairs	◯	◯	◯	◯	◯
b. Go down stairs	◯	◯	◯	◯	◯
c. Kneel on the front of your knee	◯	◯	◯	◯	◯
d. Squat	◯	◯	◯	◯	◯
e. Sit with your knee bent	◯	◯	◯	◯	◯
f. Rise from a chair	◯	◯	◯	◯	◯
g. Run staight ahead	◯	◯	◯	◯	◯
h. Jump and land on your involved leg	◯	◯	◯	◯	◯
i. Stop and start quickly	◯	◯	◯	◯	◯

FUNCTION:

How would you rate the function of your knee on a scale of 0 to 10 with 10 being normal, excellent function and 0 being the inability to perform any of your usual daily activities which may include sports?

FUNCTION PRIOR TO YOUR KNEE INJURY:
(0 = Cannot perform daily activites and 10 = No limitation in daily activities)

◯ 0 ◯ 1 ◯ 2 ◯ 3 ◯ 4 ◯ 5 ◯ 6 ◯ 7 ◯ 8 ◯ 9 ◯ 10

CURRENT FUNCTION OF YOUR KNEE:
(0 = Cannot perform daily activites and 10 = No limitation in daily activities)

◯ 0 ◯ 1 ◯ 2 ◯ 3 ◯ 4 ◯ 5 ◯ 6 ◯ 7 ◯ 8 ◯ 9 ◯ 10

Total score: ____________________

Raw Score ____________________

KOOS QOL

These questions should be answered thinking of your knee symptoms during the last week.

How often are you aware of your knee problem?

◯ Never
◯ Monthly
◯ Weekly
◯ Daily
◯ Constantly

Have you modified your life style to avoid potentially damaging activities to your knee?

◯ Not at all
◯ Mildly
◯ Moderately
◯ Severely
◯ Totally

How much are you troubled with lack of confidence in your knee?

◯ Not at all
◯ Mildly
◯ Moderately
◯ Severely
◯ Totally

In general, how much difficulty do you have with your knee?

◯ None
◯ Mild
◯ Moderate
◯ Severe
◯ Extreme

KOOS QOL Total ____________________

Adverse Events

Have you had any new health problems or symptoms, or have any of your existing health conditions or symptoms worsened since you started in the study?

◯ Yes
◯ No

Please tell us the details of this? (what was it, who did you see etc) ____________________

Have you had any issues related to your knee that has required you to see another surgeon or be admitted to hospital elsewhere?

◯ Yes
◯ No

Please tell us the details of this? (what was it, who did you see etc) ____________________
